# Physical Activity, Sedentary Behavior, and Weight Status of University Students during the COVID-19 Lockdown: A Cross-National Comparative Study

**DOI:** 10.3390/ijerph18137125

**Published:** 2021-07-03

**Authors:** Seok Tyug Tan, Chin Xuan Tan, Seok Shin Tan

**Affiliations:** 1 Department of Healthcare Professional, Faculty of Health and Life Sciences, Management and Science University, University Drive, Off Persiaran Olahraga, Seksyen 13, Shah Alam 40100, Malaysia; sttan@msu.edu.my; 2 Department of Allied Health Sciences, Faculty of Science, Universiti Tunku Abdul Rahman, Jalan Universiti, Bandar Barat, Kampar 31900, Malaysia; tancx@utar.edu.my; 3 Department of Nutrition and Dietetics, School of Health Sciences, International Medical University, Bukit Jalil, Kuala Lumpur 57000, Malaysia

**Keywords:** physical activity, sedentary behavior, weight status, university students, lockdown

## Abstract

The temporary closure of learning institutions during the COVID-19 pandemic has dramatically reduced the physical activity of students across all ages. Therefore, this study aimed to investigate the prevalence of physical inactivity and the patterns of physical activity among university students in confinement. This cross-sectional study involved 147 Malaysian students and 107 Indonesian students. Body weight before the pandemic and during the pandemic was self-reported by the respondents, and the International Physical Activity Questionnaire-Short Form (IPAQ-SF) was used to assess the physical activity and sedentary behavior of the respondents. The findings revealed that 79.6% of Malaysians and 77.6% of Indonesians were physically active during the confinement. There was no significant difference (*p* < 0.05) in the duration devoted to vigorous-intensity activity (M_Malaysian_ = 0.00 MET minutes/week and M_Indonesian_ = 480.00 MET minutes/week) and moderate-intensity activity (M_Malaysian_ = 0.00 MET minutes/week and M_Indonesian_ = 0.00 MET minutes/week) among the studied population. During the pandemic, Malaysian students (M = 1386.00 MET minutes/week) devoted a significantly higher duration to walking (M = 1386.00 MET minutes/week) and sedentary behavior (9.16 ± 4.47 h/day) than Indonesian students (M = 990.00 MET minutes/week and sedentary behavior = 7.85 ± 4.27 h/day). Overall, no significant difference was noted in the total physical activity of Malaysian and Indonesian students during the pandemic (M_Malaysian_ = 2826.00 MET minutes/week and M_Indonesian_ = 1782.00 MET minutes/week). Findings from Spearman’s rank correlation test suggested that there was a weak inverse correlation between the duration engaged in vigorous-intensity activity and weight change among the Malaysian students (r_s_ = −0.199, *p* = 0.016), after adjusting for gender and age. Overall, the closure of learning institutions and exercise facilities has further prevented individuals from complying with the WHO recommendation of moderate-to-vigorous physical activity during the period of home confinement.

## 1. Introduction

The coronavirus disease (COVID-19) is a contiguous viral disease that has infected more than 175 million people globally as of 13 June 2021 [1]. Malaysia and Indonesia are among the few countries that enforced a mandatory cordon sanitaire to curb the chain of COVID-19 transmission. During lockdown, local governments implemented several restriction measures to suppress the community spread of COVID-19. In Malaysia, the federal government closed all learning institutions and exercise facilities to maintain social distancing [2]. In the middle of March 2020, a number of higher institutions in Indonesia opted for remote learning in response to the uncontrolled outbreak in the country [3].

During this unprecedented pandemic, remote learning and working from home emerged as a new normal. Although these restrictive measures effectively curb the spread of COVID-19, they can also heighten the prevalence of physical inactivity globally. The temporary closure of learning institutions altered the daily routine of students, in which they were no longer required to travel to schools for learning purposes. This change dramatically reduced the physical activity level of students across all ages during confinement [4]. Numerous studies have investigated the impact of COVID-19 national lockdowns on the physical activity of various populations. However, to date, findings from these studies are inconsistent. For instance, a study by Castañeda-Babarro, Arbillaga-Etxarri, Gutiérrez-Santamaría, and Coca [5] reported a significant reduction in vigorous-intensity activity and walking time among healthy individuals in Spain during the lockdown. In contrast, findings from a cross-national comparative study in France and Switzerland indicated that lockdown resulted in a better engagement in moderate-intensity activity and walking, in addition to increased sedentary behavior [6]. Therefore, this study aimed to investigate the prevalence of physical inactivity and the patterns of physical activity among university students in Malaysia and Indonesia during the COVID-19 pandemic. 

## 2. Methods

### 2.1. Study Design and Population

Data collection was carried out from 15 to 31 December 2020 (during the COVID-19 pandemic outbreak) using a combination of convenience and snowball sampling approaches. Two groups of participants were enrolled in this cross-sectional study: 147 Malaysian undergraduate students from a private university in Selangor, Malaysia, and 107 Indonesian undergraduate students from a private university in Central Java, Indonesia. Malaysia and Indonesia were selected as the study populations because both countries comprise multiracial, multireligious, and multi-ethnic populations. A web-based self-administrated questionnaire was hosted on Google forms and disseminated to prospective respondents via WhatsApp, Facebook, Instagram, and TikTok. Informed consent was obtained from the respondents before answering the survey questions. The sample size was calculated using an online sample size calculator (https://select-statistics.co.uk/calculators/sample-size-calculator-two-proportions/ accessed on 26 April 2021) at a 95% confidence level, with 80% power; 18.9% of Malaysians [7] and 45.6% of Indonesians [8] were physically inactive during the COVID-19 pandemic. Therefore, a total of 45 students was required in each group for this study. 

### 2.2. Socio-Demographic Characteristics and Weight Status

Gender, age, body height (cm), body weight before the COVID-19 pandemic (kg), and current body weight (kg) were self-reported by the respondents. Body weight before the COVID-19 pandemic was recalled by the respondents. The respondents were instructed to measure their current body weight using a bathroom scale whenever possible. These factors were gathered via the following questions: “Please state your body weight (kg) before the COVID-19 pandemic outbreak happened in your country” and “With the aid of a bathroom scale, please state your current body weight (kg)”. The self-perceived weight change was deemed to be the difference between the two self-reported body weights. The magnitude of weight change during the COVID-19 pandemic was further classified into 5 categories (gained > 5 kg, gained ≤ 5 kg, sustained weight, lost > 5 kg, and lost ≤ 5 kg), because it was recently reported that the average weight gain among Southeast Asian populations was at least 5 kg [9]. Moreover, the Body Mass Index (BMI) before and during the COVID-19 pandemic was also collected in this study. The BMI was then further categorized into 4 groups (underweight, normal, overweight, and obese according to the Asian-Pacific cutoff points [10].

### 2.3. Physical Activity and Sedentary Behaviour (Sitting Time)

The International Physical Activity Questionnaire-Short Form (IPAQ-SF) was used to assess the level of physical activity and sedentary behavior of the students during the COVID-19 pandemic [11]. It comprises four domains intended to measure the average duration devoted to sedentary behavior (sitting), walking, and moderate- and vigorous-intensity activities. The weekly MET minutes were calculated by multiplying the MET factor assigned to each activity (walking = 3.3 MET, moderate-intensity activity = 4.0 MET, vigorous-intensity activity = 8.0 MET) by the duration (in minutes) and the number of days that the respective activity was performed. Total physical activity was the sum of weekly MET minutes spent on walking and moderate- and vigorous-intensity activities. The respondents were then dichotomized into two categories based on their total physical activity: physically inactive (<600 MET minutes/week) and physically active (≥600 MET minutes/week) [12].

Sedentary behavior (sitting time) was measured as a part of IPAQ-SF. All respondents were required to fill in the duration spent sitting at a desk, reading, or lying down to watch television during home confinement. This duration was further dichotomized into two categories: those with a daily sitting time of <8 h and those with a daily sitting time of ≥8 h [10]. The reliability of IPAQ-SF in this study was good (Cronbach’s alpha = 0.797).

### 2.4. Data Analysis

Data analysis was conducted using IBM SPSS version 26.0 (IBM Corp., Armonk, NY, USA). Descriptive statistics including frequency, percentage, median, and interquartile range (IQR) (25th–75th percentile), in addition to mean and standard deviation (SD), were used to describe variables where appropriate. The self-perceived weight change, daily sitting time (sedentary behavior), time devoted to vigorous- moderate-intensity activities, walking, and total physical activity were tested for normality. These variables were considered to be normally distributed if the skewness was ±2. The Mann–Whitney U test was used to determine the mean differences in the self-perceived weight change, time devoted to vigorous- and moderate-intensity activities, walking, and total physical activity of Malaysian and Indonesian students. The mean difference in daily sitting time (sedentary behavior) and BMI status of Malaysian and Indonesian students was evaluated with the Independent samples t-test. The correlations between physical activity, sedentary behavior, and self-perceived weight change were assessed with Spearman’s rank correlation test (r_s_), after adjustment for gender and age. Statistical significance was considered at *p* < 0.05. 

## 3. Results

Table 1 depicts the socio-demographic characteristics, physical activity, sedentary behavior, and self-perceived weight status of the respondents. There were 254 university students enrolled in this study, comprising 147 Malaysians and 107 Indonesians. The majority were females (72.1% Malaysians and 71.0% Indonesians), aged 22–25 years old (69.4% Malaysians) and 18–21 years old (78.5% Indonesians). Nevertheless, it is noteworthy that the mean ages were comparable among Malaysians (22.28 ± 1.45 years old) and Indonesians (21.07 ± 1.10 years old). 

The current study revealed that most of the students were physically active (≥600 MET minutes/week) during the COVID-19 home confinement (79.6% Malaysians and 77.6% Indonesians). It was noted that Indonesian students (M = 480.00 MET minutes/week) had a better engagement in vigorous-intensity activity compared to Malaysian students (M = 0.00 MET minutes/week); however, findings from the Mann–Whitney U test revealed no significant difference in the time devoted to vigorous-intensity activity among the students (*z* = −0.918, *p* = 0.359). Both Malaysian and Indonesian students recorded a median score of 0.00 in the moderate-intensity activity during the COVID-19 pandemic (*z* = −0.509, *p* = 0.611). However, Malaysian students (M = 1386.00 MET minutes/week) had a significantly higher walking duration than Indonesian students (M = 990.00 MET minutes/week) (*z* = −2.168, *p* = 0.030). In terms of total physical activity during the COVID-19 pandemic, Malaysian students had a slightly higher median score (M = 2826.00 MET minutes/week) than Indonesian students (M = 1782.00 MET minutes/week). Despite these differences, no significant difference was noted in the total physical activity of Malaysian and Indonesian students during the pandemic (*z* = −1.027, *p* = 0.304). In addition, the findings also indicated that Malaysian students (9.16 ± 4.47 h/day) spent a significantly longer duration engaged in sedentary behavior compared to Indonesian students (7.85 ± 4.27 h/day) during the home confinement (*t* = −2.360, *p* = 0.019) (Table 2).

**Figure 1:**
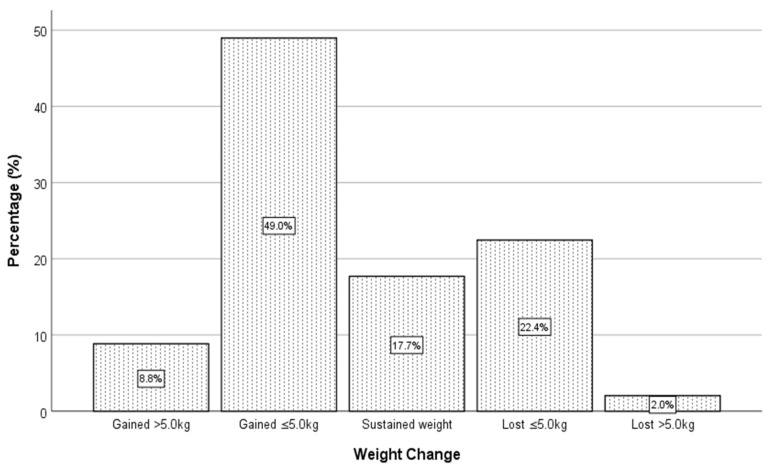
Weight change among Malaysian students during the COVID-19 pandemic.

**Figure 2:**
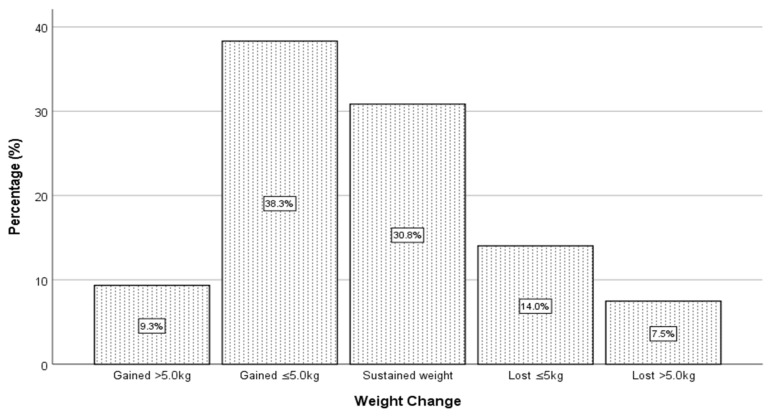
Weight change among Indonesian students during the COVID-19 pandemic.

The impact of COVID-19 home confinement on BMI and body weight was also determined in the current study. Findings from the Independent samples t-test indicated no significant difference (*t* = 1.245, *p* = 0.214) between the BMIs of Malaysian (22.33 ± 4.90) and Indonesian (21.67 ± 3.64) students before the COVID-19 pandemic. Similarly, no significant difference (*t* = 1.507, *p* = 0.133) was found between the BMIs of Malaysian (22.83 ± 4.93) and Indonesian students (22.01 ± 3.76) during the confinement. However, despite these findings, there was an increase in the BMI of Malaysian (increased by 2.24%) and Indonesian students (increased by 1.57%) during the pandemic. Of the 147 Malaysian students, slightly more than half of the respondents (57.8%) gained weight during the COVID-19 pandemic, with a median weight gain of 2.00 kg. A similar trend was also observed among Indonesian students, whereby almost half of the respondents (47.7%) experienced weight gain (M = 0.00 kg) (Table 1 and Table 2). Coincidentally, findings from the Mann–Whitney U test also revealed no significant difference in the body weight of Malaysian and Indonesian students at the time of the outbreak. It is also worth noting that the magnitude of weight gain was in the range of less than 5.0 kg (49.0% Malaysians and 38.3% Indonesians) of those who gained weight during home confinement (Figure 1 and Figure 2).

Table 3 shows the correlations between physical activity, sedentary behavior, and weight change after adjusting for gender and age. In general, no significant correlations (*p* > 0.05) were observed between the duration devoted to moderate-intensity activity and weight change, walking and weight change, total physical activity and weight change, or sedentary behavior and weight change. Furthermore, although there was a weak inverse correlation between the duration engaged in the vigorous-intensity activity and weight change among Malaysian students (r_s_ = −0.199, *p* = 0.016), such a correlation was not noticeable in its counterpart. 

## 4. Discussion

The findings of this study revealed that 20.4% of Malaysian and 22.4% of Indonesian students were physically inactive during the COVID-19 pandemic. The prevalence of physical inactivity in this study is in good agreement with the data reported in WHO [13], which stated that one in four (23%) adults do not meet the recommendations for physical activity globally. However, previous reports from national health surveys indicated that 25.1% of Malaysian adults [14] and 33.5% of Indonesian adults [15] do not adopt a physically active lifestyle. Compared to the nationally representative samples, the current population was notably more physically active during home confinement. Meyer et al. [16] also reached a similar observation. United States adults were reported to be more physically active during the lockdown than the nationally representative samples of the National Health and Nutrition Examination Survey (NHANES) in 2015–2016.

Several studies have reported the level of physical activity and duration devoted to sedentary behavior (sitting time) during the COVID-19 pandemic. In general, the prevalence of physical inactivity was 20.5% among adults older than 18 years old in the United States [16], 25.0% among adults older than 18 years old in United Kingdom [17], 26.6% among university students in Spain [4], and 29.3% among university students in Hungry [18]. In comparison, the prevalence of physical inactivity among Malaysian and Indonesian students was generally in accordance with those previously mentioned.

A recent study by Cigrovski, Škovran, Hua, Rupčić, and Knjaz [19] compared the physical activity of university students in Croatia and China during the COVID-19 lockdown (Table 4). It was found that Croatian students were more physically active than Chinese students during the global pandemic. Croatian students recorded a median total physical activity of 4259.00 MET minutes/week, with 1920.00 MET minutes/week vigorous-intensity activity, 1200.00 MET minutes/week moderate-intensity activity, and 767.25 MET minutes/week spent walking. In contrast, the median value of total physical activity among Chinese students was 1805.00 MET minutes/week, with 480.00 MET minutes/week vigorous-intensity activity, 360.00 MET minutes/week moderate-intensity activity, and 363.00 MET minutes/week walking. In comparison, the quantity of total physical activity of Malaysian students lay between those of Croatian and Chinese students. However, Indonesian students demonstrated a slightly lower median value in weekly physical activity than Chinese students. In addition, it was also noted that Indonesian and Chinese students devoted a similar duration to vigorous-intensity activity during home confinement. Thus, although the current population exhibited a lower moderate-intensity activity than Croatian and Chinese students, findings also revealed that students in this study had a better engagement in walking during the COVID-19 pandemic than previously reported. 

The time devoted to sedentary behavior (daily sitting time) is expected to increase substantially due to the need for remote learning and working as a result of the pandemic [20]. Findings in the current study indicated that Malaysian and Indonesian students spent 9.16 h/day and 7.85 h/day engaged in sedentary behavior during home confinement in response to the COVID-19 pandemic. These findings were comparable to the 8.40 h/day reported in the United States population during the pandemic [21]. It is also worth noting that the magnitude of weight gain in the current study was in the range of less than 5.0 kg. This finding was consistent with the study of Zachary et al. [22] in the United States during the COVID-19 pandemic. A number of studies have shown that individuals who were overweight/obese before the pandemic were more likely to gain additional weight during confinement [23]. It was observed that the current findings go beyond previous studies, showing that the proportions of overweight/obesity among Malaysian and Indonesian students are similar throughout the pandemic. Physical inactivity, increased sitting time due to the need for remote learning at home, and engaging in emotional eating are among the contributing factors of weight gain among students in this unprecedented pandemic [24,25]. Interestingly, emerging findings demonstrated that university students had a better engagement in physical activity, even with a substantial increase in sedentary behavior during confinement. These findings are generally in accordance with the study of van Bakel et al. [26], which reported that individuals devoted more time to walking in the pandemic. 

It is also noteworthy that the WHO recommendation for physical activity has remained unchanged during the COVID-19 pandemic [27]. The current findings demonstrated that both Malaysian and Indonesian students did not comply with the recommendation of performing at least 150 min of moderate-intensity activity or 75 min of vigorous-intensity activity per week throughout the pandemic. Interestingly, a significant proportion of Malaysian students gained weight during the pandemic, despite higher weekly physical activity than Indonesian students. This could be because the accumulated weekly physical activity of Malaysian students was solely from walking, whereas for Indonesian students it was from a combination of vigorous-intensity activity and walking. In addition, a growing body of literature also demonstrated that men are more physically active than women. In addition, the level of physical activity tends to substantially reduce with increased age [28]. Therefore, Spearman’s rank correlation coefficient analysis was conducted by controlling for the gender and age of the students. The findings indicated a weak inverse correlation between the duration devoted to vigorous-intensity activity and weight change among Malaysian students. A similar observation was obtained by Fan et al. [29], wherein the moderate-to-vigorous physical activity was correlated with lower BMI and risk of overweight/obesity. 

It must be highlighted that the current study adopted a web-based self-administrated survey, given that face-to-face data collection was not feasible during the COVID-19 pandemic. Therefore, one of the limitations naturally includes not being able to contact individuals with limited internet access. In addition, findings in the current study might not represent the physical activity levels of all university students in Malaysia and Indonesia due to the small sample size, and samples were only selected from a private university in both countries. Moreover, the self-reported anthropometric measurements may potentially be subject to recall and reporting biases. Although there was an inverse correlation between the duration engaged in the vigorous-intensity activity and weight change among Malaysian students, it must be emphasized that correlation does not imply causation. Nevertheless, a longitudinal study can be carried out to investigate the relationship between physical activity and weight fluctuation during and post COVID-19 confinement. Despite these limitations, this study was the first to compare the physical activity, sedentary behavior, and weight status among university students in Malaysia and Indonesia during the pandemic. 

## 5. Conclusions

In conclusion, findings in the current study indicated that the majority of students were more physically active, engaged in more than 8 h of sedentary behavior, and gained less than 5.0 kg body weight during the pandemic. The temporary closure of learning institutions and exercise facilities has further prevented individuals from complying with the WHO recommendation of moderate-to-vigorous activity. Nevertheless, home-based exercise should be highly encouraged and promoted throughout this unprecedented pandemic. 

## Figures and Tables

**Table 1:** Socio-demographic characteristics, physical activity, sedentary behavior, and self-perceived weight status of the respondents.

Variable	Malaysian, *N* = 147		Indonesian, *N* = 107	
	*n* (%)	Mean ± SD ^1^/Median (IQR) ^2^	*n* (%)	Mean ± SD ^1^/Median (IQR) ^2^
**Gender** Male Female	41 (27.9) 106 (72.1)	-	31 (29.0) 76 (71.0)	-
**Age**^1^ 18–21 22–25	45 (30.6) 102 (69.4)	22.28 ± 1.45	84 (78.5) 23 (21.5)	21.07 ± 1.10
**Physical activity** Physically active Physically inactive	117 (79.6) 30 (20.4)	-	83 (77.6) 24 (22.4)	-
**Sedentary behavior** <8 h/day >8 h/day	69 (46.9) 78 (53.1)	-	68 (63.6) 39 (36.4)	-
**Self-perceived weight status (kg) ^2^** Sustained weight Lost weight Gained weight	26 (17.7) 36 (24.5) 85 (57.8)	2.00 (0.00–4.00) ^a^	33 (30.8) 23 (21.5) 51 (47.7)	0.00 (0.00–3.00) ^a^
**BMI before the pandemic (kg/m^2^)** Underweight Normal Overweight Obese	29 (19.7) 68 (46.3) 18 (12.2) 32 (21.8)	22.33 ± 4.90 ^b^	18 (16.8) 56 (52.3) 12 (11.2) 21 (19.6)	21.67 ± 3.64 ^b^
**Current BMI (kg/m^2^)** Underweight Normal Overweight Obese	22 (15.0) 74 (50.3) 19 (12.9) 32 (21.8)	22.83 ± 4.93 ^b^	15 (14.0) 59 (55.1) 12 (11.2) 21 (19.6)	22.01 ± 3.76 ^b^

^1^ Mean and standard deviation (SD). ^2^ Median and interquartile range (IQR) was reported in the range of 25th to 75th percentiles. ^a^ Mean difference was tested with the Mann–Whitney U test. Significance was considered at *p* < 0.05. Different letters indicate significant differences on the same row. ^b^ Mean difference was tested with the Independent samples *t*-test. Significance was considered at *p* < 0.05. Different letters indicate significant differences on the same row. BMI: Body Mass Index.

**Table 2:** Time devoted to physical activity and sedentary behavior among university students.

Variable	Median (IQR) ^1^/Mean ± SD		*z*-Value/t-Value	*p*-Value
	Malaysian, *n* = 147	Indonesian, *n* = 107		
Vigorous-intensity activity (MET minutes) ^2^	0.00 (0.00–1440.00)	480.00 (0.00–1920.00)	−0.918	0.359
Moderate-intensity activity (MET minutes) ^2^	0.00 (0.00–720.00)	0.00 (0.00–720.00)	−0.509	0.611
Walking (MET minutes) ^2^	1386.00 (594.0–2970.0)	990.00 (396.00–2376.00)	−2.168 *	0.030
Total physical activity (MET minutes) ^2^	2826.00 (990.00–5508.00)	1782.00 (792.00–5790.00)	−1.027	0.304
Sedentary behavior (hours/day) ^3^	9.16 ± 4.47	7.85 ± 4.27	−2.360 *	0.019

^1^ IQR was reported in the range of 25th to 75th percentiles. ^2^ Mean difference was tested with the Mann–Whitney U test. ^3^ Mean difference was tested with the independent samples *t*-test. * Significance was considered at *p* < 0.05.

**Table 3:** Correlations between the studied variables after adjustment for gender and age.

Variable	Weight Change, r_s_ (*p*-Value) ^1^	
	Malaysian	Indonesian
Vigorous-intensity activity	−0.199 (0.016) *	0.095 (0.336)
Moderate-intensity activity	−0.147 (0.078)	0.038 (0.697)
Walking	−0.048 (0.565)	0.058 (0.556)
Total physical activity	−0.155 (0.062)	0.142 (0.150)
Sedentary behavior	0.008 (0.927)	0.091 (0.356)

^1^ Correlation was tested with Spearman’s rank correlation test after adjustment for gender and age. * Significance was considered at *p* < 0.05.

**Table 4:** Physical activity of Croatian and Chinese university students during the COVID-19 lockdown.

Variable	Median (IQR) ^1^	
	Croatia, ^2^ *N* = 102	China, ^2^ *N* = 120
Vigorous-intensity activity (MET minutes)	1920.00 (960.00–3840.00)	480.00 (0.00–2160.00)
Moderate-intensity activity (MET minutes)	1200.00 (600.00–1920.00)	360.00 (0.00–900.00)
Walking (MET minutes)	767.25 (462.00–2079.00)	363.00 (132.00–990.00)
Total physical activity (MET minutes)	4259.00 (2730.00–7812.00)	1805.00 (648.50–3961.00)

^1^ Interquartile range (IQR) was reported in the range of 25th to 75th percentile. ^2^ Values were obtained from Cigrovski, Škovran, Hua, Rupčić, & Knjaz [19].

## Data Availability

The data presented in this study are available within the article.
